# Global research trends in Total Body Irradiation: a bibliometric analysis

**DOI:** 10.3389/fonc.2024.1370059

**Published:** 2024-04-26

**Authors:** Mamdouh Saud Alqathami, Muhammad Ajmal Khan, Ahamed Badusha Mohamed Yoosuf

**Affiliations:** ^1^ Department of Oncology, Ministry of National Guard - Health Affairs, Riyadh, Saudi Arabia; ^2^ King Abdullah International Medical Research Center, Riyadh, Saudi Arabia; ^3^ King Saud bin Abdulaziz University for Health Sciences, Riyadh, Saudi Arabia; ^4^ Imam Abdulrahman Faisal University, Dammam, Saudi Arabia

**Keywords:** Total Body Irradiation (TBI), radiotherapy (RT), bibliometric analysis (BA), hematopoietic stem cell transplant (HSCT), bone marrow transplant (BMT)

## Abstract

**Objectives:**

This manuscript presents a bibliometric and visualization analysis of Total Body Irradiation (TBI) research, aiming to elucidate trends, gaps, and future directions in the field. This study aims to provide a comprehensive overview of the global research landscape of TBI, highlighting its key contributions, evolving trends, and potential areas for future exploration.

**Methods:**

The data for this study were extracted from the Web of Science Core Collection (WoSCC), encompassing articles published up to May 2023. The analysis included original studies, abstracts, and review articles focusing on TBI-related research. Bibliometric indicators such as total publications (TP), total citations (TC), and citations per publication (C/P) were utilized to assess the research output and impact. Visualization tools such as VOS Viewer were employed for thematic mapping and to illustrate international collaboration networks.

**Results:**

The analysis revealed a substantial body of literature, with 7,315 articles published by 2,650 institutions involving, 13,979 authors. Full-length articles were predominant, highlighting their central role in the dissemination of TBI research. The authorship pattern indicated a diverse range of scholarly influences, with both established and emerging researchers contributing significantly. The USA led in global contributions, with significant international collaborations observed. Recent research trends have focused on refining TBI treatment techniques, investigating long-term patient effects, and advancing dosimetry and biomarker studies for radiation exposure assessments.

**Conclusions:**

TBI research exhibits a dynamic and multifaceted landscape, driven by global collaboration and innovation. It highlights the clinical challenges of TBI, such as its adverse effects and the need for tailored treatments in pediatric cases. Crucially, the study also acknowledges the fundamental science underpinning TBI, including its effects on inflammatory and apoptotic pathways, DNA damage, and the varied sensitivity of cells and tissues. This dual focus enhances our understanding of TBI, guiding future research toward innovative solutions and comprehensive care.

## 1 Introduction

Total Body Irradiation (TBI), a cornerstone in the field of radiation therapy, was pioneered in the late 1950s by E.D. Thomas, a Nobel Prize recipient in Medicine (1–3). This technique, involving the administration of high-energy photon beams to the entire body, is extensively used to treat a range of both malignant and benign medical conditions. The role of TBI is particularly significant in multidisciplinary treatment approaches for managing widespread malignancies, often in combination with intensive chemotherapy. Notably, TBI is predominantly utilized in hematological malignancies such as acute lymphoblastic leukemia (ALL), acute myeloid leukemia (AML), and certain lymphomas, as part of preparative regimen for hematopoietic stem cell transplantation. While most solid tumors are treated with fractionated local radiation therapy, TBI’s application remains focused on these specific hematologic conditions due to its systemic nature (4). The key to its therapeutic application is its capacity to enable myeloablative high-dose therapy and immuno-ablative conditioning, which are essential precursors for successful hematopoietic stem cell transplantation (HSCT), involving either bone marrow or peripheral blood progenitor stem cells (4, 5).

HSCT, also referred to as bone marrow transplantation, addresses two primary categories of health conditions. The first category comprises non-malignant diseases that lead to the failure or dysfunction of bone marrow, encompassing conditions like aplastic anemia, myelodysplastic syndromes, certain immunodeficiency and genetic disorders, and hemo-globinopathies including thalassemia and sickle cell anemia. The second category targets hematopoietic malignancies, including various forms of leukemia, lymphoma, multiple myeloma, and myeloproliferative disorders (6). While achieving total eradication of the HIV reservoir is challenging, TBI, along with chemotherapy and immunosuppressive drugs, has been identified as a component contributing to the eradication of HIV-1 reservoirs (7). Also, when combined with other treatments like stem cell transplantation, TBI can lead to reductions in HIV-1 reservoirs, demonstrating its potential in combating the virus (8). Furthermore, the use of TBI in conjunction with other strategies, such as CCR5Δ32 stem cell transplantation, has shown promise in reducing HIV reservoirs and potentially achieving a cure (9, 10).

The adoption of TBI as a fundamental part of the preparative regimen for allogeneic bone marrow transplantation has marked significant advancement in this domain (11). TBI’s multifaceted role extends from eradicating diseased marrow and minimizing tumor load to facilitating crucial immunosuppression in transplants from unmatched donors and creating space for healthy donor marrow engraftment. Its effectiveness is evident in a range of conditions such as Acute Myelogenous Leukemia (AML), Acute Lymphoblastic Leukemia (ALL), and various anemias, where it is utilized as a conditioning regimen for HSCT (12).

The evolution of TBI toward a focus on enhanced immunosuppression over myeloablative cytotoxic conditioning has been a pivotal shift, notably reducing early transplant-related mortality and morbidity. This change has led to better success rates for allogeneic stem cell engraftment from both related and unrelated donors. The focus has shifted toward using the immune response from graft-versus-host disease to target minor antigens on tumor cells, improving tumor eradication (13–16). Clinical indications for TBI have expanded to include a wide spectrum of leukemia types in both adults and children, with optional applications for solid tumors in pediatric cases and ongoing clinical trials for Hodgkin’s disease and non-Hodgkin’s lymphomas (17).

Given the vast array of literature on TBI encompassing thousands of articles across various research domains, there is a pressing need for an organized approach to navigate this extensive body of knowledge. A comprehensive bibliometric analysis that goes beyond traditional reviews and meta-analyses to quantitatively and qualitatively evaluate the literature, is essential (18). Such an analysis will not only help in identifying key papers and journals, but also in understanding the contributions of different countries, institutions, and authors to this field. It will also aid in discerning prevailing research trends, hotspots, research gaps and significant advances, thereby guiding future research directions. This study aimed to perform an in-depth bibliometric analysis of TBI research, drawing upon articles published from 1942 to 2022, to map out the landscape of research in this critical area of medical science.

## 2 Materials and methods

### 2.1 Data collection strategy

The primary source of our data was the Web of Science Core Collection (WoSCC), a comprehensive database encompassing a vast array of cited scientific publications globally. We conducted an exhaustive search in WoSCC using a specified search strategy. This involved querying records as follows: (TI=(“Total body irradiation”) OR AK=(“Total body irradiation”) OR AB=(“Total body irradiation”)) OR (TI=(“Total-body irradiation”) OR AK=(“Total-body irradiation”) OR AB=(“Total-body irradiation”)). Our focus was to retrieve manuscripts written in English, encompassing the entire period from the inception of the database up to May 2023. Each record contained bibliographic information about author, affiliation, title, abstract, keywords, journal name, year of publication, volume, issue, pages, publisher, country, and total number of citations.

### 2.2 Inclusion criteria

We concentrated on original research studies, meeting abstracts, review articles, notes and letters, proceedings and other document types such as editorial content, corrections, and retractions. This choice was made to ensure a focus on the comprehensive research that contributed significantly to the field.

### 2.3 Bibliometric indicators

Our bibliometric analysis was grounded in both the quantitative and qualitative metrics. The total number of publications (TP) served as the primary quantitative measure, while total citations (TC), non-cited publications (NCP), cited publications (CP), and citations per publication (C/P) provided multifaceted qualitative assessments. These indicators offer insights into the growth, influence, and quality of research in Total Body Irradiation (19–22).

### 2.4 Data analysis techniques

The current study identified the focus of research in recent decades by using bibliometric analysis to generate a graphical mapping of keywords, which was represented by VOS. A program designed and frequently used to graphically illustrate for creating and viewing bibliometric parameters in mapping networks is called Visualization of Similarities (VOS) Viewer 1.6.17 (Leiden University, Leiden, Netherlands). Here, we employed it for mapping journals, researchers, individual publications, and relationships such as bibliographic linkages, co-citations, and co-authorships (23). The technique of counting was “full counting.” Various nodes in the graphic map stood in for authors, nations, organizations, and keywords. The matching number or reference frequency was indicated by the node size. Cooperation and co-occurrence ties were represented by the links between the nodes. The nodes and lines’ colors indicated various clusters, matching years, or average references. Basic data handling, trend plotting and descriptive statistical analysis were conducted using Microsoft Excel software.

### 2.5 Thematic mapping and international collaboration analysis

We employed correspondence analysis and clustering techniques to explore common themes and relationships between authors and institutions. A thematic map was created based on the keyword network, utilizing a clustering algorithm. Furthermore, international collaboration was assessed by examining the proportion of papers with international co-authors compared to those with national-only authorships. The most productive countries were identified based on authorship associations and the degree of international cooperation was measured (24).

### 2.6 Validation process

To ensure the accuracy and reliability of our analysis, the entire process, including data retrieval, categorization, and analysis, was verified by two authors. Through this rigorous methodology, we aimed to provide a comprehensive bibliometric and visualization analysis of Total Body Irradiation research, identifying significant trends, gaps, and future directions in the field. This approach allowed us to systematically compile and interpret data, offering valuable insights into the global research landscape of Total Body Irradiation.

## 3 Results

### 3.1 Publications and document types

In the domain of Total Body Irradiation (TBI) research, a substantial body of literature comprising 7,307 articles has been published by 59,329 authors and 197,699 citations. This extensive research underscores TBI’s significance in the scientific community. Full-length articles represented the bulk of this scholarly output, totaling 5,500 publications and amassing 187,631 citations. These figures highlight the central role of comprehensive articles in disseminating knowledge regarding TBI. Meeting abstracts, with 1,317 publications and 638 citations, underscored the importance of conferences as platforms for preliminary research dissemination and scholarly exchange. Reviews, though fewer in number (228 TP), demonstrated a significant impact with 6,683 citations, reflecting their crucial role in analyzing and synthesizing existing literature. Notes and letters, while smaller in volume (91 and 87 TP respectively), have garnered significant citations (1,637 and 553 TC), highlighting their value in facilitating rapid academic communication. Editorial materials and proceedings papers, despite lower engagement (43 and 30 TP), cater to niche audiences in the TBI field. Corrections and additions, though minimal in presence (10 and 1 TP each), play a critical role in maintaining the integrity of TBI research. A total of 707 research work has been published on the utilization of TBI for pediatric patients.

### 3.2 Publication and citation trends over time

**Figure 1:**
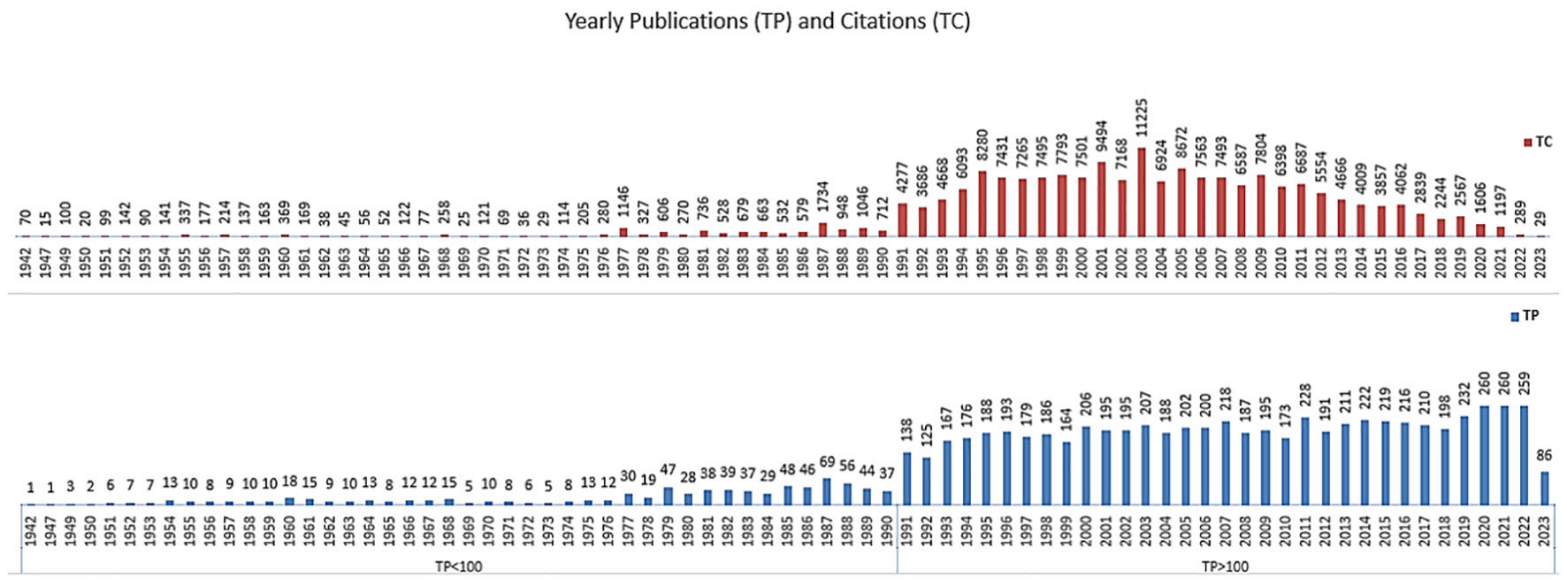
Illustrations of the yearly distribution of total number of publications (TP, in blue) and citations (TC, in red) in the field of Total Body Irradiation (TBI) from 1942 to 2023 using dual-axis bar and line graph.

**Figure 1** shows the yearly publishing and number of citations in the field of TBI. The graph is divided into two parts: the lower part shows TP<100, representing years with fewer than 100 publications, and the upper part shows TP>100, for years with more than 100 publications. The division highlights the growth in the volume of TBI research over time, with recent years seeing more than 100 publications annually, which underscore the expanding interest and ongoing research activity in this area. It was observed that the number of publications (TP) began to increase notably in the late 20th century, with a more pronounced surge seen from the 1970s onwards. This upward trend in TBI research publications continues, with fluctuations, into the 21st century, peaking in the mid-2000s. Since 2020, the TP has surpassed 250 publications three years in a row. The line graph (in red) depicts the total citations (TC) received by these publications over the same period. Citations begin to rise significantly in the 1980s, indicating a growing recognition of the work being produced in this field. A sharp increase in citations is observed from the late 1990s through the 2000s, reaching a peak in citations around 2013. Following this peak, there is a gradual decrease in the total citations, which may reflect a combination of factors, including the maturation of the field, shifts in research focus.

### 3.3 Authorship pattern

**Figure 2:**
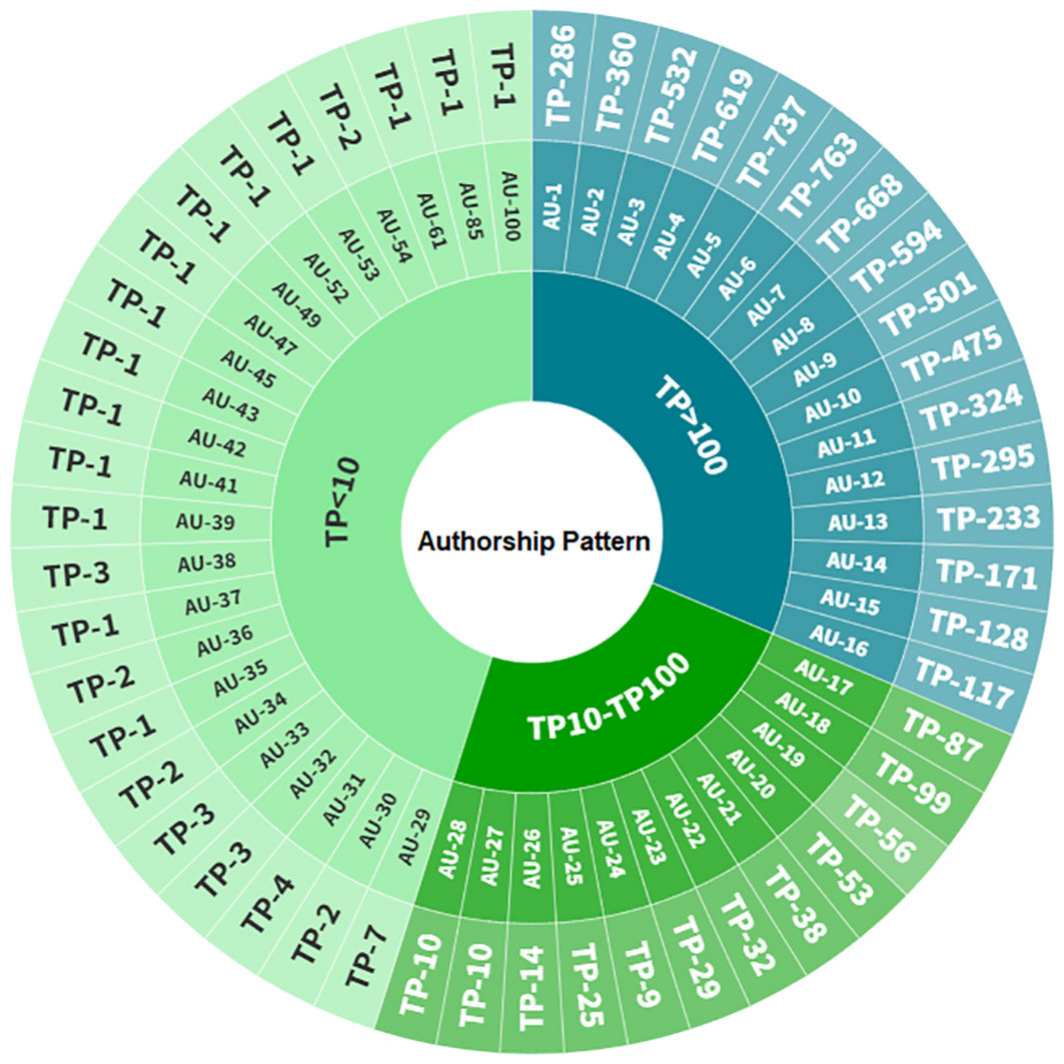
Distribution of authorship pattern in the field of Total Body Irradiation research (TP, total number of publications; AU, number of authors).

The authorship pattern in TBI research, as depicted in the donut chart (**Figure 2**), reveals the distribution of total publications (TP) and number of authors (AU) in the field of Total Body Irradiation research. The author categories are stratified based on the total number of publications (TP) attributed to each group of authors. In the TP>100 category, authors are notably prolific, with increasing TP correlating with higher total citations (TC). However, there’s a declining trend in the citation-per-paper ratio (C/P) as TP rises, hinting at a possible trade-off between volume and impact. In TP10 - TP100 group, the authors’ exhibit moderate to high TP values, with some showcasing significantly high C/P ratios, signifying impactful research despite moderate number of publications. Meanwhile, authors with TP<10 exhibit lower productivity, yet some of them demonstrate impressive C/P ratios, indicating notable impact despite a smaller number of publications. This breakdown illustrates diverse authorship patterns based on productivity and their corresponding impact within the realm of Total Body Irradiation research. Authors’ publication counts vary widely, ranging from 1 to 763, while the total citations per author span from 0 to 15,589. This diversity indicates varying levels of impact and influence among authors in the field. The average citations per publication (C/P) ranged from 1 to 209.67, further illustrating the breadth of authorship impact. This research spans a substantial period, from as early as 1942 to 2023, highlighting the field’s evolution over time. The authors have entered the TBI research landscape at different times, with start years ranging from 1942 to 2019, showcasing varied durations of involvement in TBI studies. This dataset reveals the depth and breadth of author contributions to TBI research, offering insights into scholarly influence and the field’s evolution over time.

### 3.4 Country-wise publication metrics

**Figure 3:**
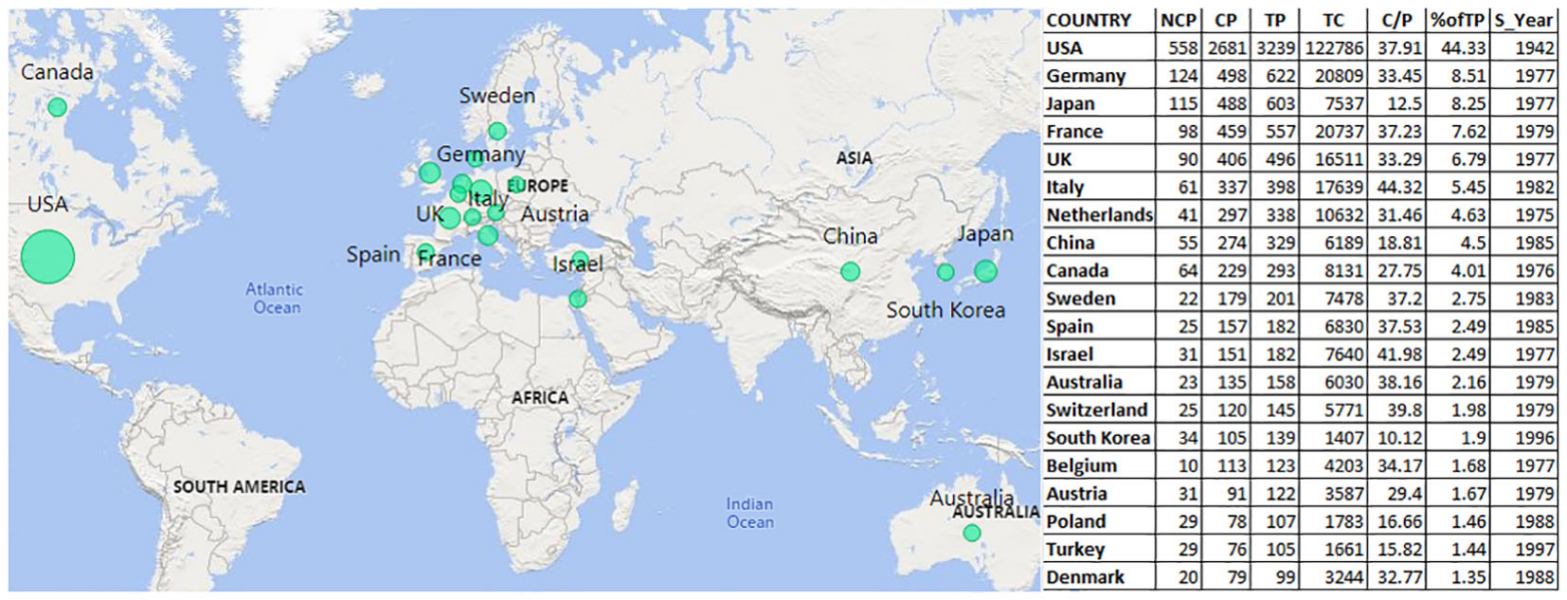
Contributions of various countries in TBI research (NCP, non-cited publications; CP, cited publications; TP, total publications; TC, total citations; C/P, citations per publication; S_year, start year).

**Figure 3** illustrates the contributions of various countries to TBI research. The USA leads, accounting for 44.33% of the total publications since 1942 and accumulated 122,786 citations. Other countries with substantial contributions include Germany (124 publications, 20,809 citations), Japan, France, and the UK, each with over 500 publications and varied citation counts. Italy, with a citation per publication (C/P) ratio of 44.32, shows substantial impact despite a lower publication count. These metrics provide a comprehensive view of the historical engagement, impact, and productivity of various countries in TBI research.

### 3.5 Author keyword burst

**Figure 4:**
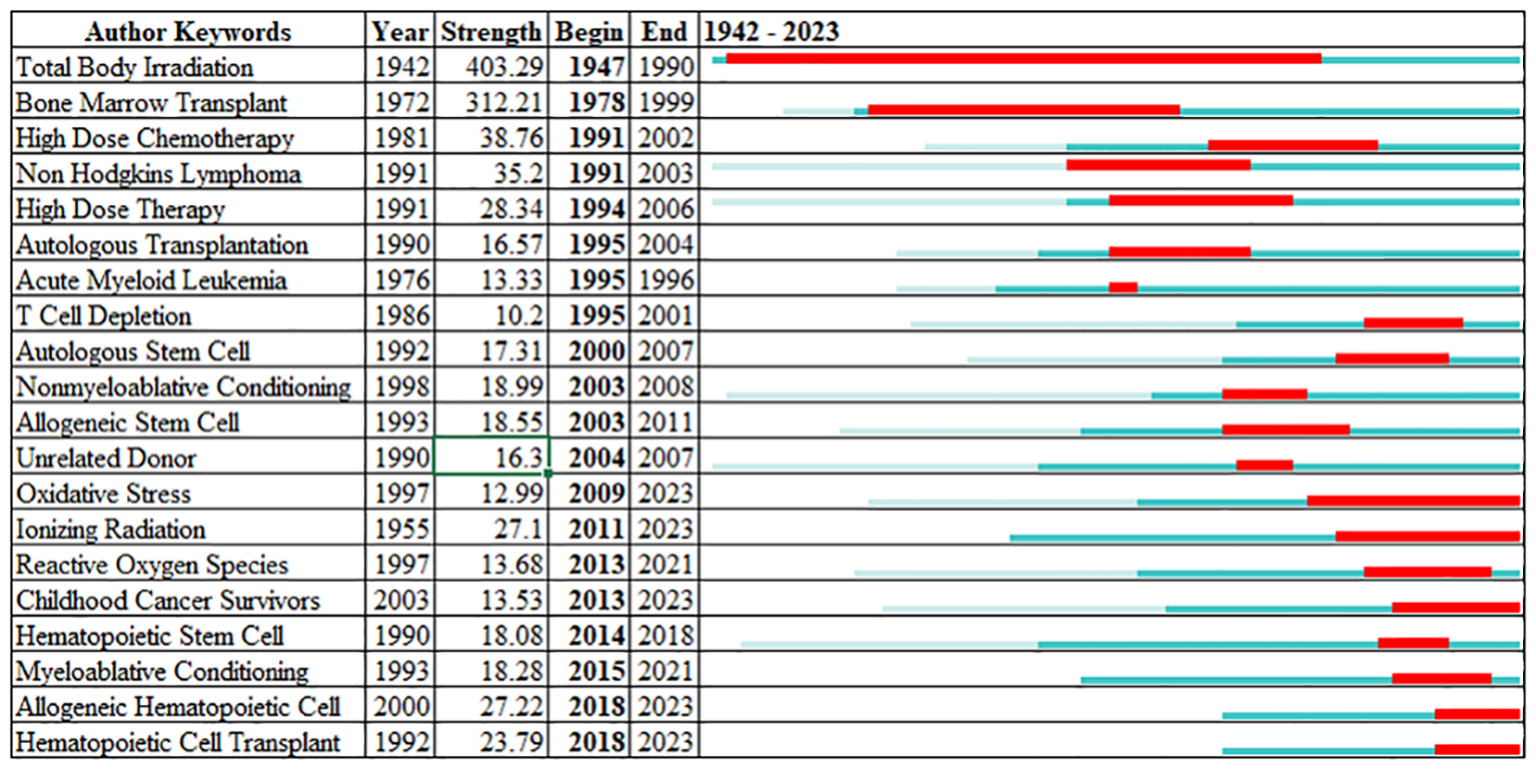
Evolution of research interests in the field of Total Body Irradiation over the period from 1942 to 2023, as reflected by the burst strength of specific keywords.

**Figure 4** demonstrates the keyword burst analysis that reflects the evolving research interest in TBI from 1942 to 2023. Early emphasis on “Total Body Irradiation” and “Bone Marrow Transplant” has shifted to recent areas like “Reactive Oxygen Species” and “Hematopoietic Cell Transplant.” The sustained interest in topics like “Autologous Transplantation” over nearly a decade highlights enduring research themes. The emergence of new keywords with ongoing burst activity signals active and evolving areas of interest, suggesting a field that is responsive to new developments and changing clinical needs. This dynamic landscape indicates the field’s responsiveness to new developments and clinical needs.

### 3.6 Research areas

**Figure 5:**
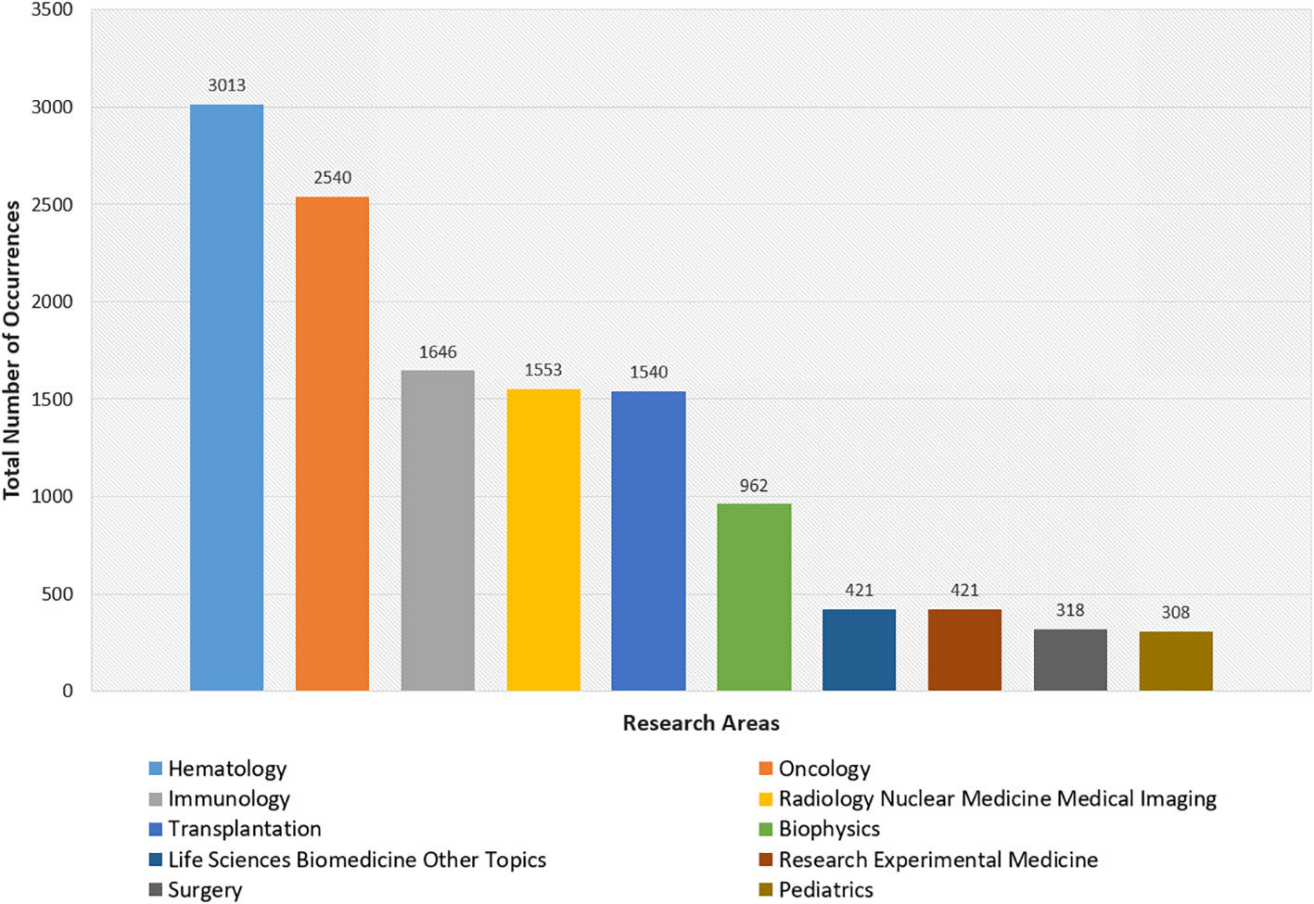
Bar chart generated from top 10 research areas related to the field of Total Body Irradiation (TBI).

**Figure 5** presents a bar chart that quantifies the research frequency within the top ten domains intersecting with TBI, revealing a hierarchical focus within the field. Hematology emerges as the most researched area, indicative of the primacy of blood-related diseases in TBI studies, while Radiology Nuclear Medicine Medical Imaging and Immunology follow, reflecting the significance of diagnostic imaging and immunological responses in TBI applications. Oncology and Transplantation are also prominently featured, underscoring TBI’s role in cancer treatment and grafting procedures, respectively. Additional areas such as Life Sciences Biomedicine Other Topics, Pediatrics, Surgery, Research Experimental Medicine, and Biophysics articulate the breadth of TBI’s interdisciplinary nature of TBI research and highlights areas of extensive scholarly focus as well as emerging disciplines within the field. Over the past decade, a substantial body of research in veterinary science has explored the use of TBI in treating canine cancers, offering a unique perspective on treatment efficacy, side effects, and the immune system’s role in cancer therapy. These studies not only advance veterinary oncology but also serve as critical models for refining TBI protocols in human medicine, underscoring the translational value of interdisciplinary research.

### 3.7 Interrelations between various research topics within the field of Total Body Irradiation

**Figure 6:**
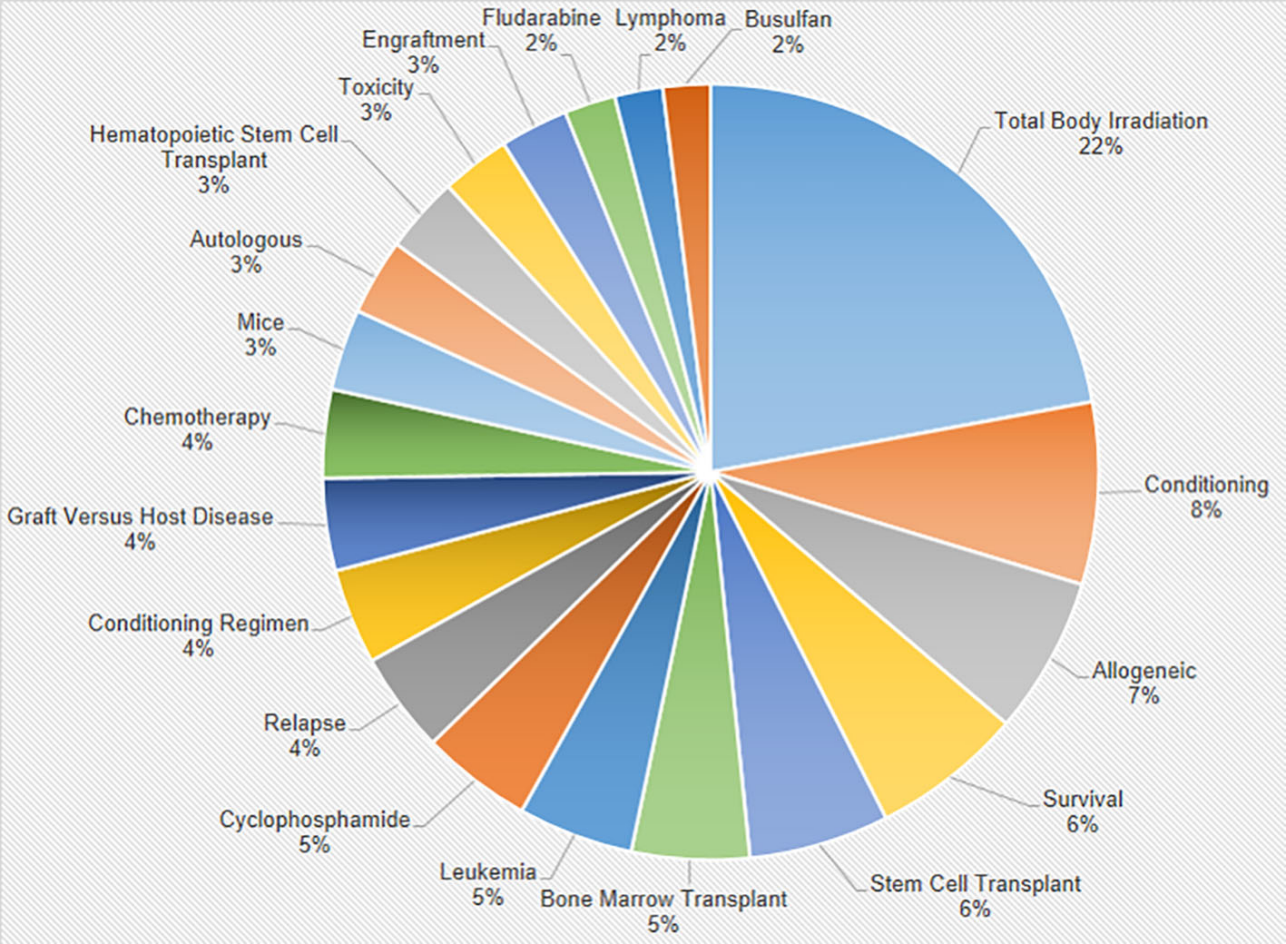
Distribution of Top 20 Author Keywords in Total Body Irradiation Research. The chart visualizes the frequency of key terms used by authors, with the segment size representing the relative prominence of each keyword within the field.

**Figure 6** is a pie chart representing the distribution of the top 20 author keywords in the field of TBI. It illustrates the scope and focus areas within TBI research, providing a visual summary of the topics that currently command the most attention and scholarly effort in the field. The chart is dominated by the term “Total Body Irradiation,” accounting for a significant 22% of the keyword frequency, which underscores its central focus in the research field. Other prominent keywords include “Conditioning” (8%), highlighting the importance of preparatory regimens for procedures such as hematopoietic stem cell transplantation; “Allogeneic” (7%) indicating a significant interest in donor-derived cell transplantations and “Survival” (6%) which suggests outcome-based research. The chart also notes substantial mentions of specific treatments and diseases such as “Leukemia” (5%), “Bone Marrow Transplant” (5%), and “Stem Cell Transplant” (6%), reflecting the therapeutic contexts in which TBI is applied. Smaller segments for “Graft Versus Host Disease” “Chemotherapy,” and “Relapse,” each at 4%, point to critical areas of concern in post-transplant outcomes and cancer treatment management. Lesser represented but still significant keywords like “Mice” (3%) indicate the use of animal models in research, while “Toxicity,” (3%) demonstrate the duality of focus on both advancing treatment strategies and understanding their potential adverse effects.

**Figure 7:**
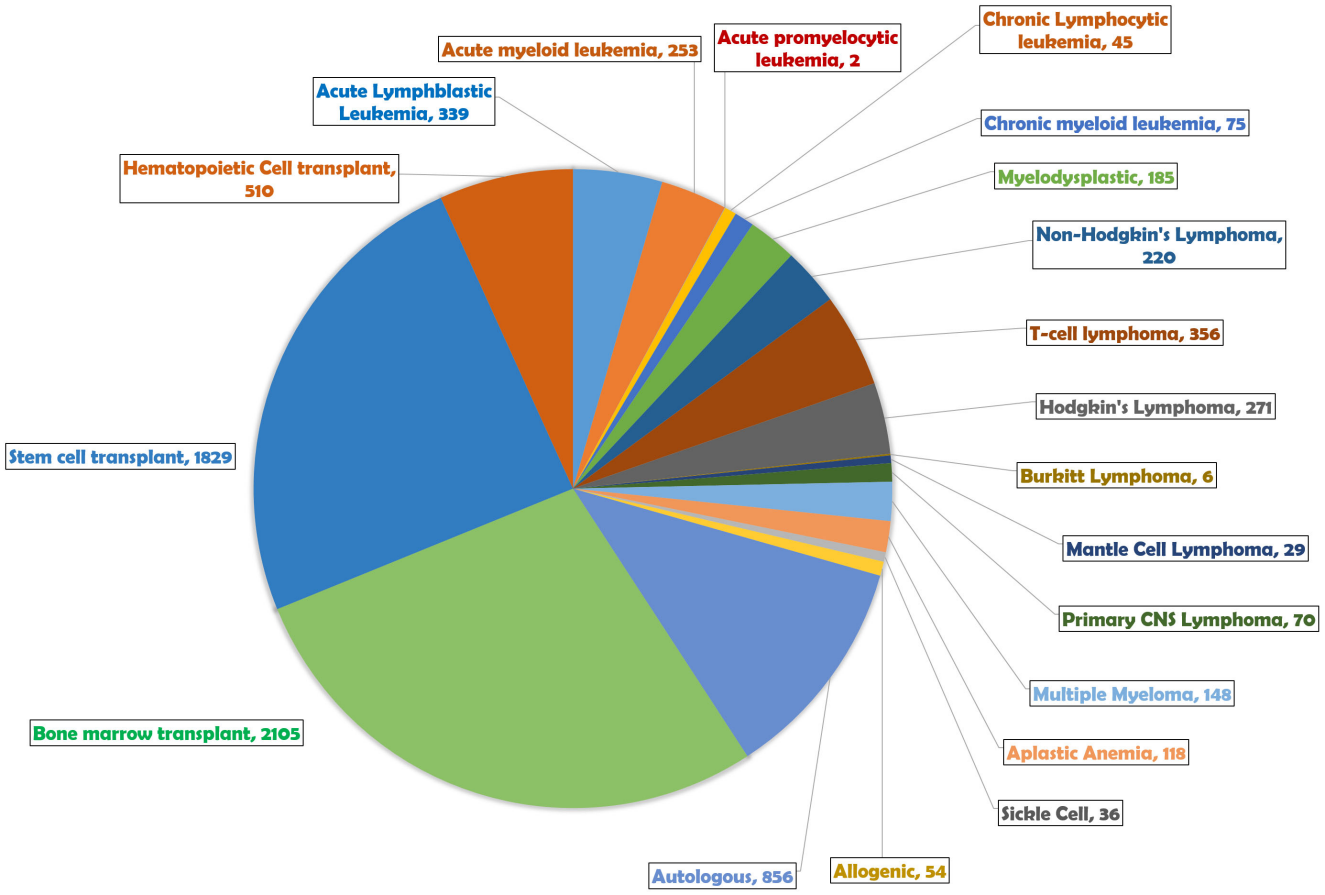
TBI indications and their respective total number of publications.

**Figure 7** summarizes the various medical indications for TBI, as evidenced by the volume of research publications over years. The chart indicates that TBI is predominantly researched in the context of bone marrow transplants (2,105 publications) and stem cell transplants (1,829 publications), highlighting these procedures as the primary applications of TBI. Hematological malignancies also feature prominently, with acute lymphoblastic leukemia (339 publications), acute myeloid leukemia (253 publications), and Hodgkin’s lymphoma (271 publications) being the most studied conditions, signifying the importance of TBI in managing these diseases. The chart additionally shows research into TBI for treating non-Hodgkin’s lymphoma (220 publications), T-cell lymphoma (356 publications), and myelodysplastic syndromes, among others, with the number of publications reflective of the clinical research interest in these areas. Less frequently indicated conditions, such as acute promyelocytic leukemia and Burkitt lymphoma, suggest niche or developing research areas within TBI application.

### 3.8 Micro-topics within the field of Total Body Irradiation

**Figure 8:**
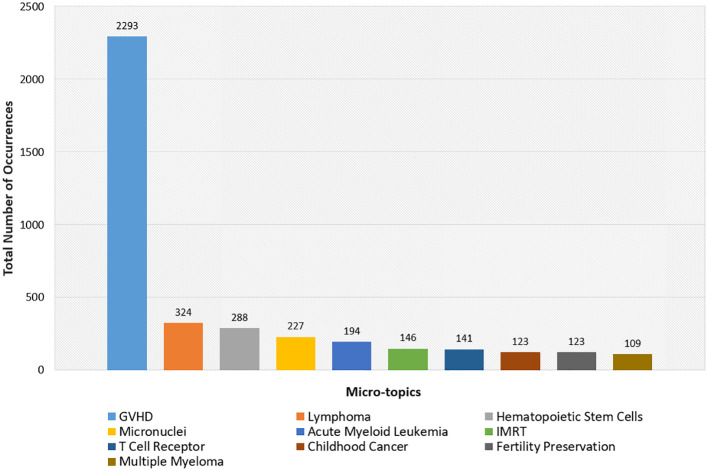
Top Ten Micro-Topics in Total Body Irradiation Research. The bar chart illustrates the frequency of key micro-topics within TBI literature depicting focal points of current research efforts.

**Figure 8** provides a bar chart representation of the top ten micro-topics within the field of TBI research, highlighting the frequency of each topic’s appearance in the scholarly literature. Graft Versus Host Disease (GVHD) is distinguished as the most frequently occurring topic, signaling its paramount importance in TBI studies. This is closely followed by notable frequencies in research on Lymphoma and Hematopoietic Stem Cells, which underscore the critical focus on TBI’s implications for hematologic conditions and stem cell regeneration. Additional areas of significant research interest include the study of Micronuclei, indicative of an investigative emphasis on cellular damage from irradiation, and Fertility Preservation, reflecting the concern for reproductive health in TBI treatment protocols. The chart further enumerates a range of specific diseases—Acute Myeloid Leukemia, Multiple Myeloma—showcasing the diverse clinical applications of TBI.

### 3.9 International collaboration network

**Figure 9:**
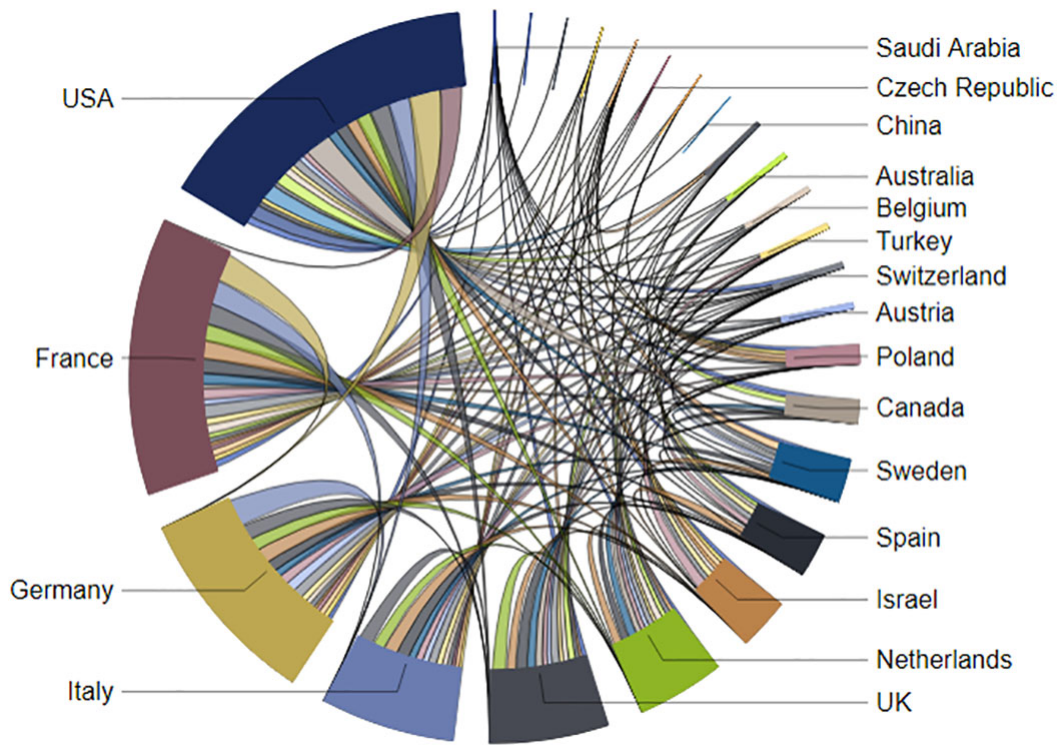
Network visualization of international collaborations in Total Body Irradiation research.

The chord diagram (**Figure 9**) portrays a vibrant network of international collaborations in Total Body Irradiation research, with the USA, France, Germany, and Italy standing out due to their larger segments, indicating they are pivotal in both output and partnerships. The thickness of the chords connecting these countries to others reflects the intensity and frequency of their research collaborations. The USA and the UK, in particular, demonstrate a multitude of connections, underscoring their roles as central hubs in the global research network. Specific collaboration patterns, possibly driven by historical ties or shared initiatives, are visible between certain countries, such as France and Germany. Overall, the intricate interconnections across the diagram reveal a rich tapestry of intercontinental cooperation, emphasizing the field’s dependency on a synergistic global scientific community to propel advancements in Total Body Irradiation.

### 3.10 Thematic analysis

**Figure 10:**
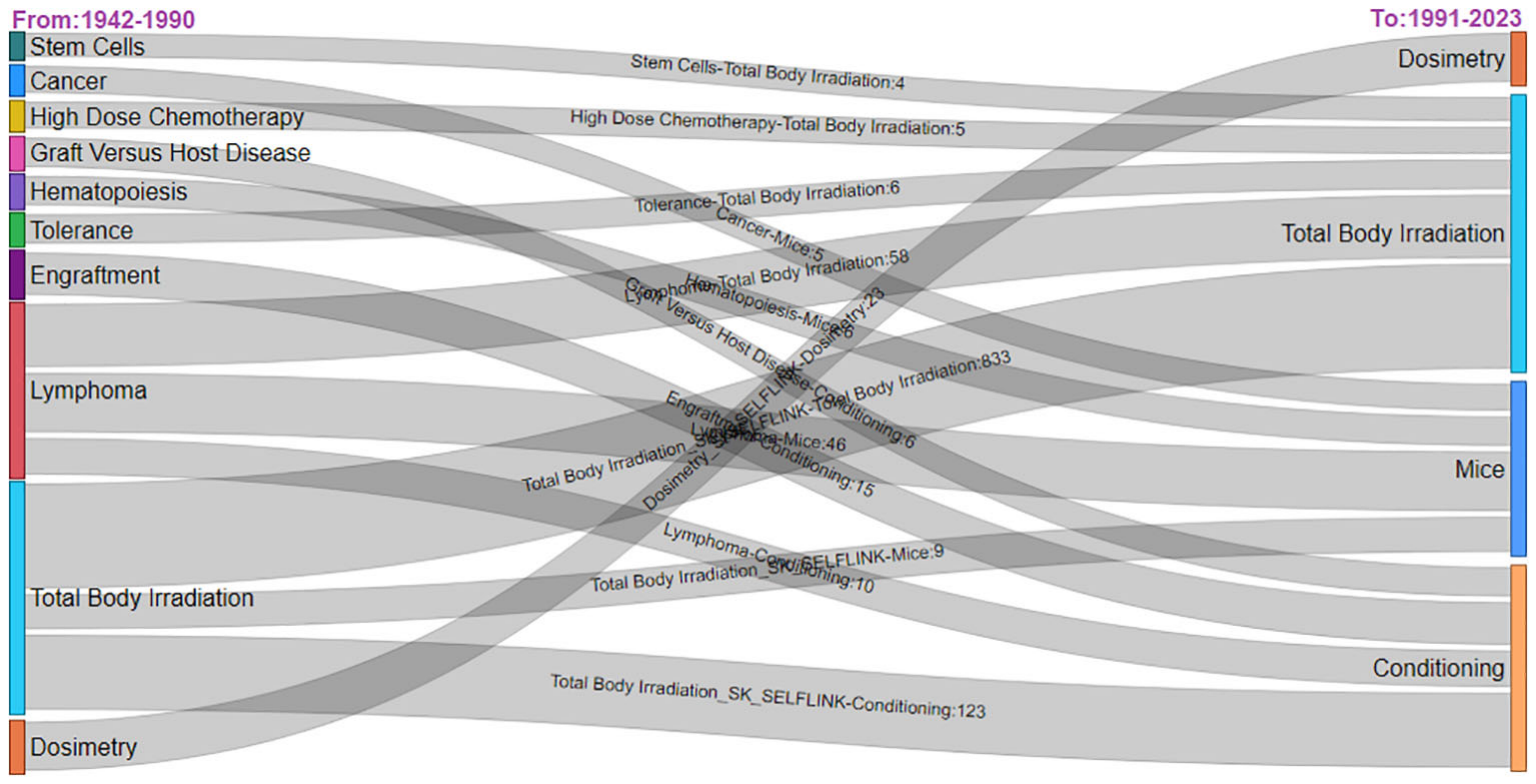
Thematic analysis in Total Body Irradiation research while also highlighting shifts in research emphasis from 1942 – 2023.

The Sankey diagram (**Figure 10**) reveals TBI research’s thematic progression, with persistent focus areas like “Total Body Irradiation” and “Cancer” and emerging themes like “Mice” in recent years, suggesting an evolution toward experimental models and novel therapeutic protocols. The interconnections between themes such as “Hematopoiesis” and “Graft Versus Host Disease” depict the intricate nature of TBI studies. The emergence of “Conditioning” as a newer theme from 1991 to 2023 reflects the development of novel therapeutic protocols. The interconnections between themes like “Hematopoiesis” and “Graft Versus Host Disease” illustrate the field’s intricate nature, with studies often spanning multiple interrelated topics. An expanded focus on “Dosimetry” suggests heightened research activity and advancements in precision measurement techniques.

### 3.11 Author’s contribution

**Figure 11:**
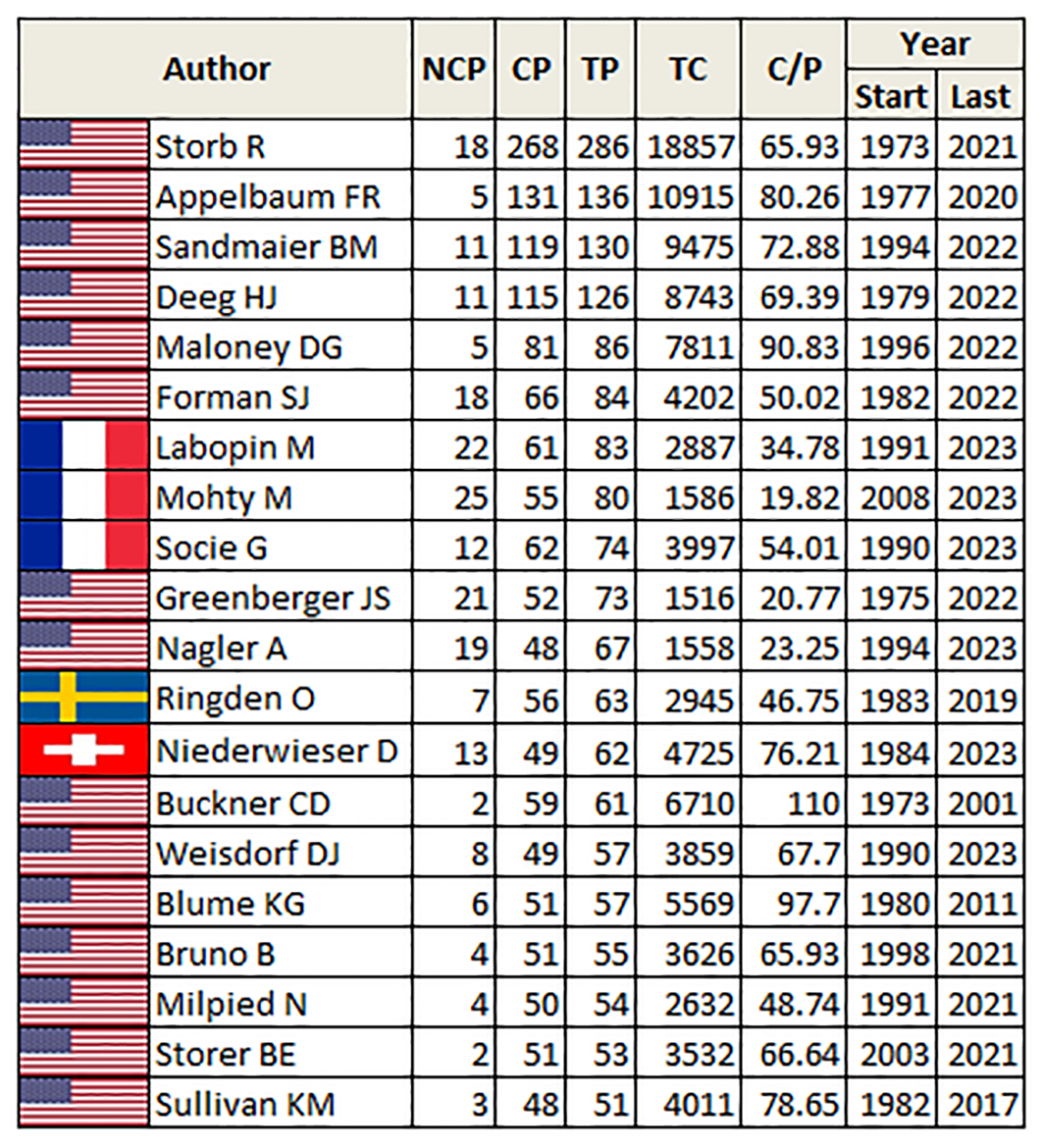
The table underscores the diverse contributions of individual authors in shaping the landscape of Total Body Irradiation research. (NCP, non-cited publications; CP, cited publications; TP, total publications; TC, total citations; C/P, citations per publication; Start, start year; Last, end year).

**Figure 11** highlights the diverse contributions of authors in TBI research, with varied publication and citation metrics reflecting a dynamic academic landscape. Prominent authors like Appelbaum FR and Sandmaier BM are noted for their high impact, while others like Storb R demonstrate a prolific and enduring contribution to the field. The range of years (Start/Last) indicates the duration of each author’s active contribution to the field. For instance, Storb R has been active from 1973 to 2021, showing a long-term commitment. Further, the NCP values show that authors like Sullivan KM have most of their work cited (low NCP), with only 3 non-cited publications, which indicates a high level of recognition within the research community.

### 3.12 Top articles

**Table 1** lists the top-cited articles that covered various aspects of cancer treatment and transplantation research, delving into intensive therapies, stem cell transplants, and treatment impacts on specific types. High-impact articles in journals like “NEW ENGL J MED” and “BLOOD” signify the influential nature of these studies in TBI research. Other investigations focus on graft-versus-host disease, secondary cancers post-irradiation, stem cell distribution, and strategies to mitigate treatment-related toxicities. These articles collectively represent significant contributions to understanding various treatment modalities, their impacts on patients, and potential avenues for improving outcomes and minimizing adverse effects in cancer therapy and transplantation.

**Table 1:** List of 20 articles with the highest citation rate in the area of total body irradiation. TI, title; TC, total citations; C/Y, citations rate per year.

Citation	TI	TC	C/Y	IF
MATTHAY KK, 1999, NEW ENGL J MED	TREATMENT OF HIGH-RISK NEUROBLASTOMA WITH INTENSIVE CHEMOTHERAPY, RADIOTHERAPY, AUTOLOGOUS BONE MARROW TRANSPLANTATION, AND 13-CIS-RETINOIC ACID (25)	1425	57	176.1
MCSWEENEY PA, 2001, BLOOD	HEMATOPOIETIC CELL TRANSPLANTATION IN OLDER PATIENTS WITH HEMATOLOGIC MALIGNANCIES: REPLACING HIGH-DOSE CYTOTOXIC THERAPY WITH GRAFT-VERSUS-TUMOR EFFECTS (26)	1100	47.83	22.1
THOMAS ED, 1977, BLOOD	100 PATIENTS WITH ACUTE-LEUKEMIA TREATED BY CHEMOTHERAPY, TOTAL-BODY IRRADIATION, AND ALLOGENEIC MARROW TRANSPLANTATION (27)	1011	21.51	22.1
DUDLEY ME, 2008, J CLIN ONCOL	ADOPTIVE CELL THERAPY FOR PATIENTS WITH METASTATIC MELANOMA: EVALUATION OF INTENSIVE MYELOABLATIVE CHEMORADIATION PREPARATIVE REGIMENS (28)	1007	62.94	50.7
CHANG JH, 2016, NAT MED	CLEARANCE OF SENESCENT CELLS BY ABT263 REJUVENATES AGED HEMATOPOIETIC STEM CELLS IN MICE (29)	963	120.38	87.2
HALL EJ, 2003, INT J RADIAT ONCOL	RADIATION-INDUCED SECOND CANCERS: THE IMPACT OF 3D-CRT AND IMRT (30)	944	44.95	8
AVERSA F, 1998, NEW ENGL J MED	TREATMENT OF HIGH-RISK ACUTE LEUKEMIA WITH T-CELL-DEPLETED STEM CELLS FROM RELATED DONORS WITH ONE FULLY MISMATCHED HLA HAPLOTYPE (31)	919	35.35	176.1
TAYLOR PA, 2002, BLOOD	THE INFUSION OF EX VIVO ACTIVATED AND EXPANDED CD4(+)CD25(+) IMMUNE REGULATORY CELLS INHIBITS GRAFT-VERSUS-HOST DISEASE LETHALITY (32)	845	38.41	22.1
CURTIS RE, 1997, NEW ENGL J MED	SOLID CANCERS AFTER BONE MARROW TRANSPLANTATION (33)	674	24.96	176.1
LAUGHLIN MJ, 2001, NEW ENGL J MED	HEMATOPOIETIC ENGRAFTMENT AND SURVIVAL IN ADULT RECIPIENTS OF UMBILICAL-CORD BLOOD FROM UNRELATED DONORS (34).	673	29.26	176.1
HILL GR, 1997, BLOOD	TOTAL BODY IRRADIATION AND ACUTE GRAFT-VERSUS-HOST DISEASE: THE ROLE OF GASTROINTESTINAL DAMAGE AND INFLAMMATORY CYTOKINES (35)	663	24.56	22.1
MATTHAY KK, 2009, J CLIN ONCOL	LONG-TERM RESULTS FOR CHILDREN WITH HIGH-RISK NEUROBLASTOMA TREATED ON A RANDOMIZED TRIAL OF MYELOABLATIVE THERAPY FOLLOWED BY 13-CIS-RETINOIC ACID: A CHILDREN’S ONCOLOGY GROUP STUDY (36)	620	41.33	50.7
SPIELBERGER R, 2004, NEW ENGL J MED	PALIFERMIN FOR ORAL MUCOSITIS AFTER INTENSIVE THERAPY FOR HEMATOLOGIC CANCERS (37)	592	29.6	176.1
BENSINGER W, 1995, J CLIN ONCOL	FACTORS THAT INFLUENCE COLLECTION AND ENGRAFTMENT OF AUTOLOGOUS PERIPHERAL-BLOOD STEM-CELLS (38)	585	20.17	50.7
GIRALT S, 2009, BIOL BLOOD MARROW TR	REDUCED-INTENSITY CONDITIONING REGIMEN WORKSHOP: DEFINING THE DOSE SPECTRUM. REPORT OF A WORKSHOP CONVENED BY THE CENTER FOR INTERNATIONAL BLOOD AND MARROW TRANSPLANT RESEARCH (39)	573	38.2	5.7
BENSINGER WI, 1995, BLOOD	TRANSPLANTATION OF ALLOGENEIC PERIPHERAL-BLOOD STEM-CELLS MOBILIZED BY RECOMBINANT HUMAN GRANULOCYTE-COLONY-STIMULATING FACTOR (40)	570	19.66	22.1
DEVINE SM, 2003, BLOOD	MESENCHYMAL STEM CELLS DISTRIBUTE TO A WIDE RANGE OF TISSUES FOLLOWING SYSTEMIC INFUSION INTO NONHUMAN PRIMATES (41)	570	27.14	22.1
AVERSA F, 2005, J CLIN ONCOL	FULL HAPLOTYPE-MISMATCHED HEMATOPOIETIC STEM-CELL TRANSPLANTATION: A PHASE II STUDY IN PATIENTS WITH ACUTE LEUKEMIA AT HIGH RISK OF RELAPSE (42)	543	28.58	50.7
BURDELYA LG, 2008, SCIENCE	AN AGONIST OF TOLL-LIKE RECEPTOR 5 HAS RADIOPROTECTIVE ACTIVITY IN MOUSE AND PRIMATE MODELS (43)	529	33.06	40.7
LAWRENCE TS, 1995, INT J RADIAT ONCOL	HEPATIC TOXICITY RESULTING FROM CANCER-TREATMENT (44)	506	17.45	8

## 4 Discussion

In this bibliometric analysis, several key findings have been observed that reflect the evolving landscape of Total Body Irradiation (TBI) research. Articles associated with research on TBI over the nine decades were analyzed from different dimensions and perspectives. The number of TBI articles has grown annually, showing rising interest in radiotherapy research.

### 4.1 Diversified research focus

The substantial volume of literature, encompassing 7,315 articles from 2,650 institutions involving 13,979 authors, underlines the extensive global interest and research efforts in TBI. The dominance of full-length articles as the primary mode of scholarly communication indicates a mature field with comprehensive research outputs. The significant number of meeting abstracts and reviews further suggests an active community engaged in ongoing discussions and evaluations of new findings. This diversity in publication types reflects a field that is both well-established and continually evolving.

### 4.2 Evolving research trends in Total Body Irradiation (TBI)

This diversity in publication type reflects a field that is both well-established and continually growing. The evolving scope of TBI research encompasses diverse aspects, from treatment techniques to clinical applications and patient impacts. For instance, Peters et al., conducted a multinational, phase III study on TBI in conditioning regimens for patients with acute lymphoblastic leukemia (ALL) undergoing HSCT, reflecting the interest in tailoring TBI protocols for specific patient groups (40). It has shown that combining TBI with chemotherapy significantly enhances event-free survival rates in pediatric patients with ALL, with rates ranging from 50-58% compared to 29-35% without TBI (44). Recent advancements in TBI protocols have seen a shift toward more personalized and targeted approaches, which have significantly impacted clinical outcomes. For instance, the use of individualized TBI protocols tailored to patient-specific factors, such as age, comorbidities, and disease stage, has allowed for better risk stratification and treatment optimization. One example is the age-adapted TBI regimen, where lower doses are employed for older patients or those with significant comorbidities to reduce toxicity while maintaining efficacy. Helical tomotherapy and novel dose fractionation strategies aim to improve efficacy while minimizing risks, showcasing a trend toward refining TBI delivery for enhanced outcomes (45, 46). This precision has been particularly beneficial in pediatric patients, where the risk of long-term developmental side effects is a significant concern. The development of techniques such as linac-based VMAT and highly conformal TBI or TMI approaches exemplify the trend toward more effective and safer radiation delivery (47, 48). This aligns with an enhanced understanding of TBI biology and physics, fostering novel delivery systems for safer and more effective radiation treatment. Patient impact studies such as by Koken & Murrer et al., on TBI practices in Belgium and the Netherlands highlight real-world applications (49). Moreover, advancements in dosimetry and biomarkers, such as the *in-vivo* dosimetry by Ganapathy et al. (2012) and radiation-responsive protein analysis by Sproull et al. (2017), are trending in recent TBI research (50, 51). These trends reflect ongoing efforts to enhance TBI’s efficacy and safety in clinical practice. Biodosimetry research (Blakely et al., 2014) and proteomic analysis for radiation exposure prediction (Sproull et al., 2017) are part of the emerging research trends (51, 52).

### 4.3 Toxicities of TBI

Despite its efficacy, TBI is associated with significant adverse effects such as endocrine deficits (53). Research on TBI’s impact on specific tissues, such as the study by Mouiseddine et al. (2007) on mesenchymal stem cells homing to radiation-injured tissues, and predictive formulas for dose-rate and survival time relationships (Chung et al., 2011), contribute to the evolving understanding of TBI (54, 55). Further, TBI is associated with other toxicities that includes late effects and secondary malignancies (53, 56). Other concerns include renal toxicity when combined with radioimmunotherapy and potential cardiotoxic effects (57, 58). Our review highlights the importance of understanding TBI-induced toxicities, including hematopoietic function impairment, as evidenced by studies on renal dysfunction, cataract formation, and cardiovascular complications, underscoring the need for comprehensive management strategies (59–61).

### 4.4 Role of TBI in pediatric cases

TBI plays a critical role in conditioning regimens for pediatric HSCT, especially in ALL treatment (62–66). Despite the potential for cognitive impairment and cardiometabolic morbidity in surviving adults, TBI remains fundamental in pediatric hematological disease treatment (67–70). The use of TBI in the treatment of extramedullary leukemia in adolescents and its significant role in achieving engraftment and leukemia eradication before HSCT are notable (71). However, balancing potential toxicities with therapeutic benefits is crucial, particularly in younger patients (72). Long-term toxicity and secondary malignancies, especially in childhood leukemia cases, have been documented (73). The practice of dose fractionation has further translated to better patient outcomes by delivering the total radiation dose in several smaller fractions rather than a single large dose. This approach has mitigated the acute side effects associated with TBI, such as nausea and fatigue, and has been associated with improved engraftment rates and reduced severity of GVHD. Preventing long-term complications of TBI in surviving children is crucial, given its association with various adverse effects. Measures to mitigate these complications include avoiding radiotherapy in young children, especially those under three years old, reducing the total radiation dose, and limiting the volume of the brain irradiated (74). Long-term results have shown that reduced-intensity conditioning regimes with moderately reduced TBI doses may be associated with lower acute morbidity and toxicity, suggesting a potential strategy to minimize adverse outcomes (75).

### 4.5 Future directions

Looking forward, the field of TBI stands at a pivotal juncture. The need for more comprehensive research to identify gaps in knowledge and explore new avenues is evident. Advances in TBI, such as helical tomotherapy, are refining treatment precision and reducing side effects. The potential of emerging trends, such as the application of biomarkers in assessing radiation exposure and the exploration of novel therapeutic approaches in radiation injuries, opens new horizons for research and clinical practice.

### 4.6 Limitations

Publications were limited to the Web of Science search engine, therefore, publications from other databases or in languages other than English may have been missed, leading to bias in citation statistics. The number of citations reported in this study may be slightly different. Several variables affect citation counts, including author, field of study, and time since publication. For instance, early publications typically had more citations. Because the key articles analyzed with the highest number of citations were published before 2013, some new publications may have been overlooked. This study used annual citation counts to compensate for the effect of publication time on the most cited articles. Further, in interpreting the citation metrics presented in this analysis, it is important to acknowledge the potential influence of various factors such as the author’s prominence, the reputation of the affiliated institution, and the extent of self-citation. These elements can skew the perceived impact or relevance of a study, suggesting that citation counts may not always be a direct reflection of research quality or significance.

## 5 Conclusion

In conclusion, the bibliometric analysis sheds light on the dynamic and multifaceted nature of TBI research. This underscores the importance of continued innovation, global collaboration, and a comprehensive approach to addressing the challenges associated with TBI. As the field progresses, it is imperative to balance advancing treatment efficacy with a deeper understanding and mitigation of potential adverse effects, particularly in vulnerable populations such as pediatric patients.

## Data availability statement

The raw data supporting the conclusions of this article will be made available by the authors, without undue reservation.

## Author contributions

MSA: Writing – review & editing, Writing – original draft, Visualization, Validation, Methodology, Investigation, Formal analysis, Conceptualization. MK: Writing – review & editing, Writing – original draft, Visualization, Formal analysis, Data curation, Conceptualization. AM: Writing – review & editing, Writing – original draft, Visualization, Validation, Supervision, Project administration, Methodology, Formal analysis, Data curation, Conceptualization.
